# Solving routing problems with pairwise synchronization constraints

**DOI:** 10.1007/s10100-018-0520-4

**Published:** 2018-02-07

**Authors:** Sophie N. Parragh, Karl F. Doerner

**Affiliations:** 1 Production and Logistics Management, Johannes Kepler University Linz, Altenberger Strasse 69, 4040 Linz, Austria; 2 Faculty of Business, Economics and Statistics, University of Vienna, Oskar-Morgenstern-Platz 1, 1090 Vienna, Austria

**Keywords:** Visit time synchronization, Metaheuristic, Routing and scheduling

## Abstract

Pairwise route synchronization constraints are commonly encountered in the field of service technician routing and scheduling and in the area of mobile care. Pairwise route synchronization refers to constraints that require that two technicians or home care workers visit the same location at exactly the same time. We consider constraints of this type in the context of the well-known vehicle routing problem with time windows and a generic service technician routing and scheduling problem. Different approaches for dealing with the problem of pairwise route synchronization are compared and several ways of integrating a synchronization component into a metaheuristic algorithm tailored to the original problems are analyzed. When applied to benchmark instances from the literature, our algorithm matches almost all available optimal values and it produces several new best results for the remaining instances.

## Introduction

This research is motivated by a problem situation commonly encountered in the area of field staff routing and scheduling. It concerns the issue of pairwise route synchronization in both space and time. Many companies plan individual daily tours for each of their field employees since most tasks require only a single staff member. However, both in the service technician field and in the area of mobile care, for some of the tasks to be completed, two staff members are necessary. One example, brought to us by a company from the service technician industry, concerns tasks that demand ladders exceeding a certain size. In this case the second staff member is necessary for both mounting and securing the ladder during the execution of the task. A second example concerns the area of mobile care. For some of the daily hygiene tasks, overweight clients have to be lifted. Also in this case a second staff member is required. Pairwise time and space route synchronization introduces timely interrelationships between all those routes serving a client requiring a synchronized visit. In this paper we evaluate several different ways to deal with such a requirement in the context of a metaheuristic algorithm.

In Sect. 2, we give a brief overview of existing contributions dealing with route synchronization issues. Thereafter, we define two generic routing problems with synchronization constraints. In Sect. 4, we describe the proposed solution framework and different approaches for incorporating the synchronization aspect into an existing metaheuristic algorithm. In Sect. 5, the different strategies for dealing with pairwise route synchronization are compared to each other and the best strategy is then used to solve available instances from the literature (Bredström and Rönnqvist 2008). Concluding remarks and directions for future research are given at the end of the paper.

## Related work

Route synchronization requirements are not only encountered in the area of mobile care or service technician routing and scheduling; they also exist in fields such as air craft fleet assignment, air borne parcel shipment, ready mixed concrete delivery, snow plowing, ship routing, person transportation, log-truck scheduling and military operations.

In the area of mobile care, route synchronization has been addressed by Eveborn et al. (2006, 2009) and Bredström and Rönnqvist (2007, 2008). While Eveborn et al. (2006) narrow the time windows of synchronized tasks to given points in time in order to guarantee synchronized visits, Eveborn et al. (2009) and Bredström and Rönnqvist (2007, 2008) use more sophisticated approaches. In Eveborn et al. (2009), synchronization time windows are handled as soft constraints and penalties are used to ensure that two staff members visit the respective client at approximately the same time. In Bredström and Rönnqvist (2007), synchronization issues are dealt with in the branching strategy, assuming that time windows, durations and traveling times are integers; and in Bredström and Rönnqvist (2008) an optimization based method is developed that handles route synchronization in terms of constraints within a mathematical model. The instances of Bredström and Rönnqvist (2008) have recently also been solved by Afifi et al. (2016), outperforming the results of Bredström and Rönnqvist (2008). Afifi et al. (2016) propose a simulated annealing based algorithm that, in every iteration, first removes a random number of customers, applies a local search procedure on the obtained partial solution and then repairs the solution using a best insertion algorithm. The algorithm prioritizes the insertion of those nodes that could not be feasibly inserted in past iterations. It keeps track of the maximum possible shift value for each inserted node, taking into account synchronization requirements, updating these values after each insertion. Due to the synchronization requirement, more than one route may be affected. In this paper, we propose several different approaches to deal with the synchronization requirement and we show their value by comparing our method to the one of Afifi et al. (2016) on the instances proposed by Bredström and Rönnqvist (2008).

Mankowska et al. (2014) consider a home health care routing and scheduling problem with temporal dependencies that also involve synchronized visits. The authors propose a matrix-based solution representation that allows for simple route evaluations in local search algorithms. The proposed heuristic methods are applied to instances with up to 300 tasks.

A more generic point of view on routing with temporal dependencies between different routes is taken by Dohn et al. (2011). They consider minimal and maximal time lags between customer visits which can also be used to consider synchronized visits. They introduce requirements of this kind into the vehicle routing problem with time windows (VRPTW) and propose a branch-and-price method that, like Bredström and Rönnqvist (2007), relies on branching on time windows. Several different master problem formulations are evaluated on instances derived from the Solomon (1987) data set with 25 and 50 customers.

In the area of field employee routing and scheduling, Li et al. (2005) propose construction heuristics and a simulated annealing approach for a manpower allocation problem. For each task to be performed, one to several workers of different qualification classes have to meet. The neighborhood operators work on job permutations which are transformed into feasible worker schedules using construction heuristics. Dohn et al. (2009) propose a branch-and-price approach for a similar manpower allocation problem, where instead of single workers, teams of workers may have to be synchronized in order to perform certain tasks. Task synchronization is taken care of in the branching scheme.

In the context of aircraft fleet assignment, the issue of schedule synchronizations is considered by Ioachim et al. (1999). The proposed algorithm is based on Dantzig–Wolfe decomposition, using column generation embedded in a branch-and-bound scheme.

Synchronization requirements are also considered in Armacost et al. (2002) and Armacost et al. (2004) in the context of air borne parcel shipments. Here, synchronization between aircraft is necessary at ramp transfers.

In ready mixed concrete delivery, synchronization issues involve the requirement of a support vehicle to assist in the unloading operations at some of the construction sites. The support vehicle has to arrive before or at the time the first vehicle delivering concrete arrives at the site. In addition, usually, the amount of concrete demanded at each site exceeds the capacities of the vehicles and once the concrete delivery has started it has to be continued (more or less) without interruption. The latter aspect introduces another synchronization requirement into the problem. Schmid et al. (2009) solve the underlying optimization problem by means of a hybrid method, relying on the notion of feasible delivery patterns. Delivery patterns are produced by a variable neighborhood search algorithm which is used to explore the neighborhood of a given feasible solution. A multi-commodity flow model is iteratively solved on the pool of generated patterns to obtain improved solutions. The same problem is also considered by Schmid et al. (2010); it is solved by means of a hybrid method combining variable neighborhood search and very large neighborhood search.

Also in snow plowing operations, synchronization between vehicles is necessary on specific arcs in the street network (Perrier et al. 2008; Salazar-Aguilar et al. 2012). These arcs usually represent multilane street segments which require one or more vehicles operating in parallel, an aspect which is referred to as tandem service by Perrier et al. (2008).

Temporal synchronization requirements in the context of a ship routing and scheduling problem, which is related to the pickup and delivery problem with time windows, are considered by Andersson et al. (2011) and Stålhane et al. (2015). The former propose an arc flow based as well as path-flow models where the set of paths are generated a priori. The latter propose a branch-and-price algorithm. In contrast to several other problems involving temporal synchronization requirements, temporally dependent visits may be performed by the same ship.

Rousseau et al. (2013) use constraint programming to deal with synchronization issues in the context of a vehicle routing problem in a dynamic environment.

In the field of log-truck scheduling, from the forest areas to the mills, synchronization of resources is required. Trucks and log-loaders have to be synchronized at the forest area. In the paper of El Hachemi et al. (2013) a weekly planning horizon and also the inventories at the woodmills are taken into consideration. At each mill and each forest location a single log loader is located. If the loader is busy when a truck arrives it has to wait and this causes costs. The problem is decomposed into a tactical and an operational problem. In the tactical problem the destinations of the trucks are determined. In the operational problem the routing problem is solved with a constrained based local search procedure. The sequencing part is handled by an iterated local search method, the synchronized scheduling with a greedy procedure. The algorithm was tested on two real world case studies. The method developed by Bredström and Rönnqvist (2008) was also applied to a log truck scheduling problem.

Also in military aircraft mission planning synchronization is required (Quttineh et al. 2013). Due to the nature of the attack, two aircraft need to rendezvous at the target, they need to be synchronized in both space and time. One aircraft is launching a guided weapon. The other is illuminating the target. A mathematical programming model is presented and tested on instances which could be solved to optimality using the proposed model.

In the field of person transportation, different variants of the dial-a-ride problem with transfers exist. The general dial-a-ride problem with transfers is considered by Schönberger (2017) and Masson et al. (2014). A special variant of the dial-a-ride problem with transfers is introduced in Reinhardt et al. (2013). In this paper the transportation of persons with reduced mobility at airports between terminals is addressed. It is a multi-mode transportation problem, where different modes of intra-terminal and inter-terminal transportation are used. In Bögl et al. (2015) the problem of school pupil routing with transfers is studied. In this problem a situation is considered where some of the school pupils have to change buses. The fact that transfers between vehicles are considered introduces a synchronization requirement into the considered problems. In all these works metaheuristics based on local search with specific operators for the synchronization requirement are designed.

For a survey on further synchronization issues in the context of vehicle routing problems we refer to Drexl (2012).

## Problem definitions

In this paper we consider two routing problems with pairwise synchronization constraints. The first problem is a generic problem based on the well-known VRPTW. The second problem is motivated by a real-world problem encountered by an Austrian infrastructure and maintenance service provider: the service technician routing and scheduling problem (STRSP). In the following, we denote the first problem as VRPTW with pairwise synchronization (VRPTWPS) and the second problem as STRSP with pairwise synchronization (STRSPPS).

Both problems can be formulated on a directed graph $G = (V,A)$ where *V* is the set of vertices and *A* the set of arcs. A given number of locations *n* has to be served by a given number of routes *m*. For ease of exposition later on, two vertices are used to denote origin and destination depot of each route *k*, although they all refer to the same physical location. Thus, the set $D_o= \left\{ n+1\ldots n+m \right\}$ contains the origin depot of each route $k \in K = \left\{ 1\ldots m \right\}$ and the set $D_d = \left\{ n+m+1 \ldots n+2m \right\}$ each route’s destination depot; the set of all depots is denoted as $D = D_o \cup D_d$. Depending on the problem, vehicles traveling along the different routes are either associated with a capacity *C* or the technicians serving the routes are associated with qualification levels for different skills; in the latter case, $p^k_f$ denotes the qualification level of technician *k* for skill *f*. The set of customer or task vertices is referred to as $V'$. Each vertex $i \in V'$ is associated with a time window $[e_i,l_i]$ and a service time $s_i$. Depending on the context, each vertex $i \in V'$ is further associated with either a demand $d_i$ or with minimum qualification level requirements $q_{if}$; the set of qualifications is denoted as *Q*. Customer or task locations demanding synchronized visits are modeled as two distinct vertices. Then, $S \subset V'$ denotes the set of vertex pairs to be synchronized. Furthermore, since outsourcing is an option in the service technician routing and scheduling problem, as defined in Kovacs et al. (2012), not serving a vertex $i \in V'$ incurs a cost $o_i$. These costs are set to very high values if serving a vertex is not optional, that is, in the case of the VRPTWSP. Traversing arc (*i*, *j*) costs $c_{ij}$ and takes $t_{ij}$ time units. The vertex set thus becomes $V = V' \cup D$ and the arc set $A = \left\{ (i,j) | i \in V\!\setminus \!\!D_d, j \in V\!\setminus \!\!D_o, i\ne j \right\}$. Using binary variables $x_{ij}^k$ to indicate whether arc (*i*, *j*) is used by route *k*, and continuous variables $B_{i}$, giving the beginning of service time at vertex *i*, we are now able to formulate the VRPTWPS:

1 $$\begin{aligned} \min \sum _{k \in K } \sum _{(i,j) \in A} x_{ij}^k c_{ij} + \sum _{i \in V'} o_i \left( 1 - \sum _{k \in K } \sum _{j|(i,j) \in A} x_{ij}^k \right) \end{aligned}$$

subject to:

2 $$\begin{aligned}&\sum _{k \in K } \sum _{j|(i,j) \in A} x_{ij}^k \le 1 \quad \forall i \in V', \end{aligned}$$

3 $$\begin{aligned}&\sum _{j|(j,i) \in A} x_{ji}^k - \sum _{j|(i,j) \in A} x_{ij}^k = 0 \quad \forall i \in V', k \in K, \end{aligned}$$

4 $$\begin{aligned}&\sum _{j |(n+k,j) \in A} x_{n+k,j}^k = 1 \quad \forall k \in K, \end{aligned}$$

5 $$\begin{aligned}&\sum _{j | (j,n+m+k) \in A} x_{j,n+m+k}^k = 1 \quad \forall k \in K, \end{aligned}$$

6 $$\begin{aligned}&\sum _{i \in V'} d_i \sum _{j | (i,j) \in A} x_{ij}^k \le C \quad \forall k \in K, \end{aligned}$$

7 $$\begin{aligned}&B_j \ge (B_i + s_i + t_{ij}) x_{ij}^k \quad \forall (i,j) \in A, k \in K, \end{aligned}$$

8 $$\begin{aligned}&e_i \le B_i \le l_i \quad \forall i \in V, \end{aligned}$$

9 $$\begin{aligned}&B_i = B_j \quad \forall (i,j) \in S, \end{aligned}$$

10 $$\begin{aligned}&x_{ij}^k \in \left\{ 0,1 \right\} \quad \forall (i,j) \in A, k \in K. \end{aligned}$$

The objective function (1) minimizes total routing and outsourcing costs. Constraints (2) and (3) make sure that each vertex is visited at most once and that, if it is visited, it is entered and left. Constraints (4) and (5) ensure that each route starts from the origin depot and returns to the destination depot. Constraints (6) guarantee that the vehicle capacity is not exceeded. Constraints (7) set the beginning of service time variables and constraints (8) impose bounds in terms of time windows on these variables. Finally, Eq. (9) guarantee that tasks requiring synchronized visits are visited by two routes at exactly the same time. Since all vertices are associated with a service time $s_i > 0$, no further constraints are necessary to enforce that two routes visit the synchronized vertex at the same time, i.e. the two vertices used to represent a customer or task requiring a synchronized visit cannot be scheduled on the same route.

Replacing constraints (6) by

11 $$\begin{aligned} q_{if} \sum _{j | (i,j) \in A} x_{ij}^k&\le p^k_f \quad \forall i \in V', k \in K, f \in Q. \end{aligned}$$

which make sure that the respective technician disposes of at least the qualification level demanded by the respective task, results in the STRSPPS. We note that these constraints boil down to a given vehicle *k* being compatible with vertex *i* if the required qualification level is met. This can be achieved by setting $x_{ij}^{k}$ to 0 whenever *i* and *k* are incompatible.

## Solution framework

We propose an adaptive large neighborhood search (ALNS) algorithm to solve the VRPTWPS and the STRSPPS. The algorithm is very similar to the ALNS developed in Kovacs et al. (2012) for a STRSP without synchronization requirements. In the following we first briefly describe the ALNS and we then propose several different approaches for dealing with synchronization requirements.

### Adaptive large neighborhood search

In general, ALNS works as follows. Starting with a first feasible solution, in every iteration, a destroy and a repair operator are employed to the current incumbent solution, hopefully yielding a solution of improved quality. In ALNS, as first proposed by Ropke and Pisinger (2006), a set of destroy and a set of repair operators are used and an adaptive scheme guides their selection. While in the original version destroy and repair operators are selected and remunerated separately for good performance, Kovacs et al. (2012) use operator pairs.

The destroy operators employed in Kovacs et al. (2012) and also in this paper are random removal, worst removal, related removal, and cluster removal. The random removal operator removes a certain number of randomly chosen customers or tasks from their routes. The worst removal operator removes customers or tasks which require long detours. The related removal operator removes customers or tasks which are related in terms of location and visit time. The cluster removal operator chooses routes, partitions each route into two subsets, and removes the customers or tasks of one of the two subsets.

The employed repair operators are a greedy insertion, a sequential insertion (Solomon 1987), and several regret insertion heuristics. The greedy insertion heuristic inserts, in every iteration, the customer or task whose insertion increases the objective function value the least, considering all routes in parallel. The sequential insertion heuristic fills one route after the other. The regret insertion heuristics calculate regret values considering the best insertion positions on different routes and they iteratively insert the task or customer with the highest regret value at its best position. All insertion heuristics are used in their deterministic as well as in a randomized fashion.

The initial solution is constructed with the greedy repair heuristic. Since we assume a large enough number of vehicles in the case of the VRPTWPS, and the outsourcing option in the case of the STRSPPS, a feasible solution can always be generated from scratch. In addition, the ALNS is embedded into a simulated annealing framework (Kirkpatrick et al. 1983). Thus also deteriorating solutions are accepted with a certain probability. For further details we refer the interested reader to Kovacs et al. (2012).

### Approaches to deal with visit synchronization

We propose several different ways to address the visit time synchronization requirement. The idea of the first two approaches is to keep the available ALNS rather untouched. This means that within the ALNS, synchronization is guaranteed by assigning a given visit time to those tasks that require synchronized visits; and to add a component, which could be considered a local search component, that tries to improve this timing for the different synchronized tasks. The third approach modifies certain elements of the ALNS, mainly the request insertion scheme, in order to identify good visit times for synchronized visits.

#### Approach A: individual synchronized timing optimization

The aim of approach A is to improve the positions of the synchronized tasks within their routes, one at a time. During the execution of the ALNS, time windows of synchronized tasks are narrowed to points in time. This approach guarantees that both vehicles arrive at the same time at the synchronized customer/task and it allows us to use the ALNS of Kovacs et al. (2012) without further modifications. In order to improve this point in time, we propose to integrate the following enumerative procedure, which is called once for each task requiring a synchronized visit as described in Algorithm 1:
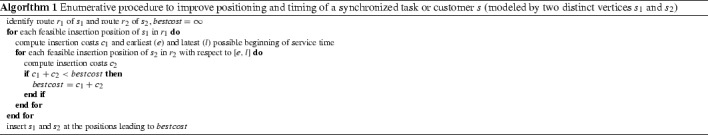


The procedure is employed with a certain probability, regardless of the quality of the newly generated solution. To control how often it is used we use an adaptive mechanism, similar to the one used to identify the next destroy and repair operator pair. Let $w_{sync}$ and $w_{nosync}$ denote the weights for using the synchronization component in the current iteration and for not using it, respectively. Based on these weights a roulette wheel selection mechanism decides if the enumerative procedure is employed in the current iteration. The weights are initialized as follows:

$$\begin{aligned} w_{sync} = \frac{100}{i_{freq}} \quad w_{nosync} = 100 - \frac{100}{i_{freq}}, \end{aligned}$$

where $i_{freq}$ is a user defined parameter that indicates how often the synchronization component should be called within the first 100 iterations. We chose 100 iterations since this corresponds to the frequency used to update the weights of the destroy and repair operator pairs. During the same time segment, also the synchronization component accumulates points for good performance. We use score parameters $\psi _{sync}$ and $\psi _{nosync}$ and a counter parameter $n_{sync}$. All three are initialized with 0 at the beginning of every new segment of 100 iterations. The counter parameter counts how often the synchronization component was called during the last segment, and, following Ropke and Pisinger (2006), $\psi _{sync}$ and $\psi _{nosync}$ are increased in the following cases:

- if a new best solution is identified ($\sigma = 33$)
- if the new solution was not visited before and improves the incumbent solution ($\sigma = 9$)
- if the new solution was not visited before and is accepted although it is worse than the incumbent ($\sigma = 13$)

that is, in the above cases, if the synchronization component was called, $\psi _{sync} := \psi _{sync} + \sigma$ and $\psi _{nosync} := \psi _{nosync} + \sigma$ otherwise. The values used to update the scores are based on Kovacs et al. (2012) and Ropke and Pisinger (2006). Then, at the end of every time segment, the weights are updated as follows:

$$\begin{aligned}&w_{sync} = 0.99 \, w_{sync} + 0.01 \, \frac{\psi _{sync}}{\max (1,n_{sync})}\\&w_{nosync} = 0.99 \, w_{nosync} + 0.01 \, \frac{\psi _{nosync}}{\max (1,100-n_{sync})}. \end{aligned}$$

Besides the enumerative approach to improve the insertion as well as the timing of a synchronized task, we also used a dynamic programming algorithm to solve the synchronization subproblem and we modeled it as a set partitioning type problem which can be solved by a linear programming solver. They are described in Appendix A.1. They are not competitive in terms of run time. However, even if they are not competitive for the current setting they may be of interest for solving problems with synchronization requirements which are not limited to pairs of routes.

#### Approach B: global synchronized timing optimization

Approach A takes a very local view. Therefore, we also developed an approach that does not only improve the insertion position of one synchronized task at a time but of all currently inserted synchronized tasks. In order to do so, we set up a mixed integer program (it extends the CGit approach for pairwise route synchronization given in Appendix A.1.2). It uses the following notation:

**Table:** 

$\Omega$	set of all feasible routes.
*R*	set of route indices of those routes that are in the current feasible solution.
$\Omega _k$	set of all feasible alternative routes of original route *k*.
$D_r$	pairs (*i*, *j*) of synchronized tasks that are scheduled along route *r* such that *i* precedes *j* and no other synchronized task is scheduled between *i* and *j*.
$\Omega _{r(i)}$	set of all feasible routes containing task *i*.
$c_r$	routing costs of route *r*.
$b_{ir}$	earliest beginning of service time of task *i* on route *r*.
$w_{rij}$	waiting time between *i* and *j* on route *r*.
$f_{ri}$	forward time slack of task *i* on route *r*.
*S*	pairs of synchronized tasks.
$y_r$	binary decision variable equal to 1 if route *r* is selected and 0 otherwise.
$F_i$	continuous decision variable giving by how much the beginning of service at *i* should be shifted.

With this notation, we are now able to formulate the global synchronized timing optimization problem.

12 $$\begin{aligned} \min \sum _{r \in \Omega } c_r y_r \end{aligned}$$

subject to:

13 $$\begin{aligned}&\sum _{r \in \Omega _k} y_{r} = 1 \quad \forall k \in R, \end{aligned}$$

14 $$\begin{aligned}&\sum _{r \in \Omega _{r(i)}} y_{r} b_{ri} + F_{i} = \sum _{r \in \Omega _{r(j)}} y_{r} b_{rj} + F_{j} \quad \forall \left\{ i,j \right\} \in S \end{aligned}$$

15 $$\begin{aligned}&0 \le F_i \le f_{ri} + (1-y_{r}) M \quad \forall r \in \Omega , i \in r, \end{aligned}$$

16 $$\begin{aligned}&\max (0, F_{i} - w_{rij}) - (1-y_{r}) M \le F_{j} \quad \forall r \in \Omega , (i, j) \in D_r, \end{aligned}$$

17 $$\begin{aligned}&y_{r} \in \left\{ 0,1 \right\} \quad \forall r \in \Omega . \end{aligned}$$

Objective function (12) minimizes the total routing costs. Constraints (13) make sure that exactly one route is chosen from the set of all feasible routes that could replace a given route in the current solution. Equation (14) make sure that synchronized tasks are visited at exactly the same time on both routes serving them. Constraints (15) make sure that the beginning of service of a task *i* is not shifted by more than the available slack and constraints (16) make sure that shifted beginning of service times are correctly propagated along each route.

In order to fill the pool of all possible routes $\Omega$, we take each route part of the current solution that serves at least one synchronized task and enumerate all possible routes such that the order of non-synchronized tasks remains fixed but one route is generated for each possible insertion position of each synchronized task. Then, the above model is solved on this set of routes and in the case where the new solution is better than the current solution, the new solution replaces the current solution.

In order to avoid the generation of too many routes and thus too long computation times, we set artificial time windows around synchronized tasks. The width of these time windows depends on the length of the routes. The longer the routes, the shorter the width of the time windows. We apply approach B only to new best solutions during the execution of the ALNS.

#### Approach C: adaptive time windows

Approaches A and B only improve the position and timing of synchronized tasks within their routes and once a complete solution has been generated. Our third approach, approach C, tries to improve the timing of tasks requiring synchronization during the construction of a new solution, at the time the task is inserted: every time a synchronized customer or task *s*, modeled as two vertices $s_1$ and $s_2$, is not visited by any route, i.e. both $s_1$ and $s_2$ have been removed from their routes, its time window is reset to its original values. Only when either $s_1$ or $s_2$ is re-inserted, it is narrowed again to a point in time. Let us assume that $s_1$ has been inserted and $s_2$ is still in the pool of removed customers or tasks. Then, this point in time can be chosen from the range of feasible visit time values for $s_1$. In order to do so, the earliest beginning of service time for $s_1$ has to be computed as well as by how much it can be shifted such that no other customer’s or task’s time window is violated, i.e. we compute the forward time slack of $s_1$ as described in Savelsbergh (1992). We choose the beginning of service of $s_1$ randomly in this time interval and narrow the time window of the synchronized task *s*, i.e. $s_1$ and $s_2$, to this point in time. The drawback of this approach is that the removal as well as the insertion routine in the original ALNS have to be adapted (time windows have to be re-opened and the ALNS has to check if the synchronized customer or task was removed from both of its routes, i.e. $s_1$ and $s_2$ have been removed). Furthermore, it also requires additional updates in the repair heuristics since previously feasible insertion positions of $s_2$ may no longer be feasible.

## Computational experiments

All algorithmic components are implemented in C++. MIPs are solved using CPLEX 12.51. All experiments are carried out on Xeon CPUs at 2.5 GHz; we only use one CPU per run and, in order to allow for fair comparisons, CPLEX is restricted to one thread. All average values on a per instances level are average values over five random runs.

### Test instances

In order to test the above defined hybridization strategies, we use two types of data sets. The first one is the well known VRPTW data set of Solomon (1987). The second one is based on the VRPTW data set and has been modified in Kovacs et al. (2012) for a STRSP: the capacity constraints of the vehicles were removed and skill-level requirements for each task as well as a set of technicians with different qualification levels were introduced. Following Kovacs et al. (2012), we do not use the outsourcing costs given in the instance file but we resort to the following term: $200 + \beta _i^{1.5}$ with $\beta _i = \sum _{f \in Q} q_{if}$. In both types of data sets synchronized customers or tasks are incorporated as suggested in Rousseau et al. (2013), that is, every tenth customer or task is assumed to demand a synchronized visit, these are $\left\{ 10, 20, \ldots , 100 \right\}$, if 0 denotes the depot. In the case of the VRPTW instances, we assume that two vehicles are needed for loading purposes but the entire load is only loaded into one of the two vehicles. Thus, if again *s* denotes the synchronized customer, modeled as two customers $s_1$ and $s_2$, the demand of $s_1$ is equal to the one of *s* while the demand of $s_2$ is 0. In a similar way, we associate $s_1$ with the original skill level requirements and we do not associate $s_2$ with any skill level requirements in the case of the STRSP based instances.

In addition to the above, we also solve the instances of Bredström and Rönnqvist (2008). New best results on these instances have recently been published by Afifi et al. (2016). The data set consists of 10 base instances and 5 different time window settings for each of these base instances. They are labeled as follows: F (fixed point in time), S (small time window), M (medium sized time window), L (large time window), A (no time window). In each instance, 10% of the tasks require a synchronized visit. Furthermore, in each instance a 9-h planning horizon is assumed. This means that in order to obtain the correct values the time values given in the instance files have to be multiplied by nine divided by the provided time horizon.

**Table 1:** Comparison of approaches on VRPTW based instances: average solution values

$i_{freq}$	Fixed	Appr. B	Appr. A			Appr. A, B	Appr C	Appr. A, B, C
	-	-	10	25	100	10	–	10
C1	1251.81	1219.02	1201.93	1207.70	1213.39	1201.22	1145.94	**1145**.**08**
C2	940.01	907.46	891.42	893.01	894.90	888.55	876.47	**876**.**30**
R1	1388.09	1367.28	1357.87	1359.32	1361.14	1358.12	**1345**.**51**	1345.99
R2	1076.01	1055.10	1029.60	1030.84	1030.55	**1029**.**24**	1032.58	1029.27
RC1	1621.71	1565.86	1556.84	1562.10	1563.05	1543.27	1524.91	**1524**.**16**
RC2	1280.88	1258.89	**1220**.**50**	1221.11	1223.07	1220.82	1224.15	1220.99
Avg.	1259.75	1228.94	1209.70	1212.35	1214.35	1206.87	1191.59	**1190**.**30**

Five runs per instance, average values per instance class, best values bold faced

### Evaluation of the different synchronization approaches

In order to identify the best setting and to understand the value of the different approaches to deal with the synchronization requirement, we only use the VRPTW based instances and we perform the following experiments. We first solve the instances without any improvement with respect to the timing of the tasks requiring synchronized visits. In a second step, we apply approach A as described above and in combination with approach B, whereas approach B is only used to improve the timing of tasks requiring synchronization whenever a new best solution is found. In the case of approach A, different settings for $i_{freq}$ are tested, namely $\{10,25,100\}$. Furthermore, we also use approach C (opening and closing the time windows at removal and insertion) without any of the other approaches and together with the best approach identified in the previous set of experiments. A summary of these results is reported in Table 1. The first column gives the name of the instance class (we report average solution values per Solomon instance class). In all subsequent columns we report the respective average values for the different approaches. Column one reports results without any improvements in terms of the timing of tasks requiring a synchronized visit, i.e. the point of time of these tasks is set in the very beginning of the algorithm and it is not changed or improved later on. Then we provide results for approach B (global optimization every time a new best solution is found). Approach A does pairwise improvements and it is applied to new solutions with a certain frequency $i_{freq}$. As mentioned above, we report results for $i_{freq} \in \{10,25,100\}$. Setting $i_{freq} = 10$ in which the component is used most often gives the best results. Therefore, this setting is also used in all subsequent experiments with approach A. First we combine approaches A and B. Then, we test approach C alone (opening and closing time windows at removal and insertion). Finally, we combine all three approaches (column “Appr. A, B, C”). Overall this setting obtains the best results. It is, however, only slightly better than applying Appr. C alone. For instance classes C1, C2 and RC1, the combination of all approaches is the best one. For R1 approach C obtains the best average solution value. For R2, the best setting is the combination of approaches A and B, and for RC2 approach A performs best. In terms of solution times, all approaches require less than 1 min on average. Only in the case of instance class R2, average values of about 1.5 min are reached in combination with approach B.

Given these results, we apply the following three approaches also to the STRSP based instances: approach A with $i_{freq}=10$, approach C, and approaches A, B, C together. Table 2 gives the respective results in comparison to the fixed point in time setting, providing the same information as Table 1, i.e. average solution values per instance class.

**Table 2:** Comparison of approaches on STRSP based instances: average solution values

	Fixed	Appr. A	Appr. C	Appr. A, B, C
C1	1410.17	1352.62	**1314.44**	1319.19
C2	1212.59	1181.48	1165.84	**1164.79**
R1	1680.88	**1646.43**	1650.74	1647.00
R2	1399.72	1360.17	**1347.90**	1348.30
RC1	1804.89	1764.01	**1726.32**	1728.11
RC2	1614.39	1535.88	**1524.31**	1524.60
Avg.	1520.44	1473.43	**1454.92**	1455.33

Five runs per instance, average values per instance class, best values bold faced

In the case of the STRSPPS, it can be observed that the best method is approach C. It obtains the lowest average solution value across all instances, closely followed by the combination of all three approaches. When looking at individual instance classes, the combination of all three approaches is better in the case of instance classes C2 and R1. However, approach A performs even better in the case of instance class R1. In terms of run times, all approaches require less than 35 s on average.

We also compare the best average value per instance class to the average solution values obtained for the fixed point in time setting (i.e., no improvements on the initial timing of synchronized tasks). Improvements in terms of solution quality of between 2 and 6.8% can be obtained in case of the STRSPPS and of between 3 and 8.5% in case of the VRPTWPS.

Summarizing these results, we observe that, as expected, there is value in investing in improving the timing of tasks requiring synchronization. We also observe that approach C (opening and closing time windows at removal and insertion) has the largest impact on the solution quality. Adding approaches A and B on top of C does not always lead to additional improvements. In the case of the VRPTWPS, it leads to improvements for instance classes C1, C2, RC1 and RC2. In the case of the STRSPPS, the picture is different. Here it only leads to additional improvements for instance classes C2 and R1. In the case of instance classes C1 and RC1, it even leads to a slight deterioration in solution quality.

Although it is not entirely clear if only approach C or C in combination with A and B should be used, we used the combination of all three approaches in a comparison to optimal solutions. For this purpose, we implemented the previously described models in C++ using concert technology, CPLEX as solver engine and a run time limit of one hour. Since only few of the 100 customer/task instances could be solved to optimality, we also solved instances of reduced size, considering the first 25 and 50 customers of the VRPTWPS instances and the first 50 tasks of the STRSPPS instances. All instances that we were able to solve to optimality are reported in Table 3. We refrain from reporting lower bounds for unsolved instances since the obtained bounds are in most cases too weak to derive any conclusions regarding the quality of the obtained upper bounds. Table 3 provides the following information: the name of the original instance, the considered size (“*n*”), the average (“Avg.”) and best solution values (“Best”) of our method, their respective percentage deviations from the optimal values (“%”), and the average run time in seconds (column “Time(s)”).

**Table 3:** Comparison of our method (Appr. A, B, C) to optimal values

Instance	*n*	Opt.	Avg	(%)	Time (s)	Best	(%)
C101	25	235.34	235.34	0.00	1.97	235.34	0.00
C101	50	475.43	475.43	0.00	8.52	475.43	0.00
C101	100	1160.81	1160.80	0.00	23.27	1160.80	0.00
C105	25	235.34	235.34	0.00	2.52	235.34	0.00
C105	50	475.41	475.42	0.00	9.20	475.41	0.00
C106	25	235.34	235.34	0.00	1.93	235.34	0.00
C107	25	235.34	235.34	0.00	2.40	235.34	0.00
C201	25	245.77	245.77	0.00	3.13	245.77	0.00
C201	50	473.96	473.96	0.00	10.68	473.96	0.00
C201	100	877.59	878.34	0.09	32.42	877.59	0.00
R101	25	713.62	713.62	0.00	2.16	713.62	0.00
R101	50	1194.84	1197.79	0.25	6.72	1194.84	0.00
RC101	25	501.95	501.95	0.00	2.51	501.95	0.00
VRPTWPS avg.				0.03			0.00
C101_5x4_noTeam	50	475.43	475.65	0.05	10.09	475.43	0.00
C101_5x4_noTeam	100	1254.44	1284.34	2.38	31.19	1279.19	1.97
C101_6x6_noTeam	50	475.43	475.52	0.02	9.33	475.43	0.00
C101_6x6_noTeam	100	1202.14	1230.01	2.32	29.55	1217.15	1.25
C101_7x4_noTeam	50	594.14	600.00	0.99	8.77	594.14	0.00
C101_7x4_noTeam	100	1494.38	1503.03	0.58	25.40	1494.38	0.00
C201_5x4_noTeam	50	603.18	603.18	0.00	8.33	603.18	0.00
C201_5x4_noTeam	100	1225.12	1242.99	1.46	30.90	1229.99	0.40
C201_6x6_noTeam	50	473.96	473.96	0.00	8.90	473.96	0.00
C201_6x6_noTeam	100	991.66	1003.45	1.19	32.66	994.35	0.27
C201_7x4_noTeam	50	638.67	641.82	0.49	7.57	638.67	0.00
C201_7x4_noTeam	100	1333.78	1364.05	2.27	30.51	1333.78	0.00
R101_5x4_noTeam	50	1194.84	1197.63	0.23	10.13	1194.84	0.00
R101_5x4_noTeam	100	1790.98	1813.90	1.28	26.87	1808.11	0.96
R101_6x6_noTeam	50	1194.84	1194.84	0.00	10.57	1194.84	0.00
R101_6x6_noTeam	100	1790.79	1811.18	1.14	28.59	1806.02	0.85
R101_7x4_noTeam	50	1237.84	1241.13	0.27	9.79	1237.84	0.00
R101_7x4_noTeam	100	1880.77	1932.82	2.77	27.53	1908.84	1.49
R201_5x4_noTeam	50	947.10	951.82	0.50	7.90	950.41	0.35
R201_6x6_noTeam	50	916.53	927.19	1.16	8.40	922.93	0.70
R201_7x4_noTeam	50	1001.40	1007.76	0.64	8.96	1007.76	0.64
STRSPPS avg.				0.94			0.42

**Table 4:** Comparison of our method (Appr. A, B, C) to the optimal values (Opt.), the matheuristic of Bredström and Rönnqvist (2008) [BR08] and the simulated annealing algorithm of Afifi et al. (2016) [ADM16] on instances of Bredström and Rönnqvist (2008)

Instance	*n*	Opt.	BR08	ADM16	BKS$^\mathrm{c}$	Avg.	(%)	Time (s)	Best	(%)
1F	20	5.13$^\mathrm{a}$	5.13	–	5.13	5.13	0.00	0.98	5.13	0.00
1S	20	3.55$^\mathrm{a}$	3.55	3.55	3.55	3.55	0.00	1.08	3.55	0.00
1M	20	3.55$^\mathrm{b}$	3.55	3.55	3.55	3.55	0.00	1.08	3.55	0.00
1L	20	3.39$^\mathrm{b}$	3.39	3.39	3.39	3.39	0.00	1.18	3.39	0.00
1A	20	–	3.16	–	3.16	2.95	$-$ 6.65	1.20	2.95	$-$ 6.65
2F	20	4.98$^\mathrm{a}$	4.98	–	4.98	4.98	0.00	1.13	4.98	0.00
2S	20	4.27$^\mathrm{a}$	4.27	4.27	4.27	4.27	0.00	1.06	4.27	0.00
2M	20	3.58$^\mathrm{b}$	3.58	3.58	3.58	3.58	0.00	1.14	3.58	0.00
2L	20	3.42$^\mathrm{b}$	3.42	3.42	3.42	3.42	0.00	1.10	3.42	0.00
2A	20	–	3.34	–	3.34	2.88	$-$ 13.77	1.21	2.88	$-$ 13.77
3F	20	5.19$^\mathrm{a}$	5.19	–	5.19	5.19	0.00	0.93	5.19	0.00
3S	20	3.63$^\mathrm{a}$	3.63	3.63	3.63	3.63	0.00	1.02	3.63	0.00
3M	20	3.33$^\mathrm{b}$	3.33	3.33	3.33	3.33	0.00	1.17	3.33	0.00
3L	20	3.29$^\mathrm{b}$	3.29	3.29	3.29	3.29	0.00	1.09	3.29	0.00
3A	20	–	3.10	–	3.10	2.74	$-$ 11.61	1.04	2.74	$-$ 11.61
4F	20	7.21$^\mathrm{a}$	7.21	–	7.21	7.21	0.00	1.01	7.21	0.00
4S	20	6.14$^\mathrm{a}$	6.14	6.14	6.14	6.14	0.00	0.91	6.14	0.00
4M	20	5.67$^\mathrm{b}$	5.75	5.67	5.67	5.67	0.00	0.97	5.67	0.00
4L	20	5.13$^\mathrm{b}$	5.30	5.13	5.13	5.15	0.39	1.36	5.13	0.00
4A	20	–	4.91	–	4.91	4.29	$-$ 12.63	1.23	4.29	$-$ 12.63
5F	20	5.37$^\mathrm{a}$	5.37	–	5.37	5.37	0.00	1.15	5.37	0.00
5S	20	3.93$^\mathrm{a}$	3.93	3.93	3.93	3.93	0.00	1.30	3.93	0.00
5M	20	3.53$^\mathrm{a}$	3.53	3.53	3.53	3.53	0.00	1.13	3.53	0.00
5L	20	3.34$^\mathrm{b}$	3.34	3.34	3.34	3.34	0.00	1.12	3.34	0.00
5A	20	–	3.26	–	3.26	2.81	$-$ 13.80	1.20	2.81	$-$ 13.80
6F	50	14.45	–	–	14.45	14.49	0.28	11.96	14.46	0.07
6S	50	8.14$^\mathrm{b}$	13.69	8.14	8.14	8.14	0.00	13.62	8.14	0.00
6M	50	–	12.80	7.70	7.70	7.72	0.26	9.03	7.71	0.13
6L	50	7.14$^\mathrm{b}$	11.87	7.14	7.14	7.14	0.00	16.40	7.14	0.00
6A	50	–	11.88	–	11.88	5.99	$-$ 49.58	16.60	5.95	$-$ 49.92
7F	50	13.02	–	–	13.02	13.02	0.00	7.56	13.02	0.00
7S	50	8.39$^\mathrm{b}$	15.06	8.39	8.39	8.39	0.00	13.69	8.39	0.00
7M	50	–	13.45	7.48	7.48	7.48	0.00	11.78	7.48	0.00
7L	50	–	11.52	6.88	6.88	6.90	0.29	18.69	6.88	0.00
7A	50	–	12.41	–	12.41	5.72	$-$ 53.91	13.29	5.71	$-$ 53.99
8F	50	34.94	–	–	34.94	34.94	0.00	9.72	34.94	0.00
8S	50	9.54$^\mathrm{b}$	–	9.54	9.54	9.71	1.78	16.48	9.54	0.00
8M	50	8.54$^\mathrm{b}$	–	8.54	8.54	8.57	0.35	15.16	8.54	0.00
8L	50	–	15.16	8.00	8.00	8.07	0.88	15.19	8.03	0.37
8A	50	–	13.01	–	13.01	6.60	$-$ 49.27	20.58	6.52	$-$ 49.88
9F	80	43.48	–	–	43.48	43.60	0.28	20.21	43.55	0.16
9S	80	–	–	11.93	11.93	12.14	1.76	51.69	12.07	1.17
9M	80	–	–	10.92	10.92	11.02	0.92	37.12	10.96	0.37
9L	80	–	20.68	10.49	10.49	10.59	0.95	40.01	10.55	0.57
9A	80	–	22.89	–	22.89	8.59	$-$ 62.47	44.08	8.51	$-$ 62.82
10F	80	12.08	–	–	12.08	12.19	0.91	38.77	12.14	0.50
10S	80	–	16.24	8.60	8.60	8.61	0.12	48.69	8.55	$-$ 0.58
10M	80	–	15.33	7.62	7.62	7.70	1.05	72.30	7.67	0.66
10L	80	–	17.61	7.75	7.75	7.42	$-$ 4.26	77.02	7.38	$-$ 4.77
10A	80	–	17.59	–	17.59	6.39	$-$ 63.67	59.93	6.31	$-$ 64.13

$^\mathrm{a}$As reported in Bredström and Rönnqvist (2008)

$^\mathrm{b}$As reported in Afifi et al. (2016)

$^\mathrm{c}$Min of Opt., BR08 and ADM16

Table 3 shows that we match the optimal solution values for all VRPTWPS instances that can be solved to optimality. The picture is quite different for the STRSPPS instances. Here, we are able to solve more instances to optimality. Apparently, they are more tightly constrained due to the available incompatibilities between tasks and technicians. We are still able to match the optimal values for a majority of the instances. However, for instances C101_5x4_noTeam, C101_6x6_noTeam, and R101_7x4_noTeam with 100 tasks, the gap remains above 1%, even for the best solutions out of five random runs. Overall this comparison shows that we are able to obtain solutions that either match the optimal values or are very close to the optimal values, except for the three instances indicated above. We assume that the reason is that they are more tightly constrained than the remaining instances.

### Results for benchmark instances

Finally, we have also applied our algorithm using the combination of all three approaches (A, B, and C) to available benchmark instances from the literature (Bredström and Rönnqvist 2008) with 20–80 tasks. Bredström and Rönnqvist (2008) report results for all time window settings for instances 1–5 and for some of the time window settings for instances 6–10. Afifi et al. (2016) report results for all instances and time window settings S, M, and L. For some of the instances optimal solution values are available. In addition, we were able to solve all previously unsolved F instances to optimality. F indicates that all time windows are narrowed to a given point in time. We compare the results of our heuristic method to the best existing heuristic results or, where known, to the optimal solution values, minimizing the total travel time. These results are reported in Table 4. It contains the following information for each instance: the instance size (*n*), in terms of the number of tasks, the optimal values where known (Opt.), the best results published by Bredström and Rönnqvist (2008) and by Afifi et al. (2016) (if no result was published for the respective instance this is indicated by a “–”), the best known values (column “BKS”), and the average and best results of our method (columns “Avg.” and “Best”) as well as the percentage deviations from the best known values (“%”), and the average run time in seconds (column “Time (s)”).

We observe that our method obtains several new best results and that it matches almost all available optimal values. We note, however, that the method of Afifi et al. (2016) is faster than ours, taking at most 18.32 s of computation time.

## Conclusions

In this paper, we have combined a large neighborhood search algorithm with three different approaches to deal with tasks which require visit time synchronization. The first approach is applied with a certain frequency to new solutions encountered during the execution of the ALNS. It tries to iteratively improve the timing of one task requiring a synchronized visit at a time. The second approach takes a more global view. It tries to simultaneously improve the timing of all synchronized tasks. Since this is more time consuming, it is only applied whenever a new best solution is found. The third approach integrates the timing of synchronized tasks into the removal and insertion routine of the LNS. We have evaluated different combinations of these approaches and we have found that the largest impact on solution quality is achieved by the third approach. For the VRPTW with pairwise route synchronization constraints, the best method combines all three approaches. For the STRSP with pairwise route synchronization constraints, the third approach is the best one, very closely followed by the combination of all three approaches. When applied to benchmark instances from the literature, our method obtains several new best solutions and it matches almost all known optimal values. Finally, we have also shown that considerable improvements in terms of solution quality are possible when the timing of tasks requiring synchronized visits is improved during the course of the algorithm. In this paper, we have only investigated problem settings where tasks require up to two persons to be completed and where travel and service times are considered to be deterministic. Future research will involve the consideration of stochastic travel times in combination with synchronization requirements and it should address settings in which more than two persons are necessary to perform a task.

## Figures and Tables

**Fig. 1:**
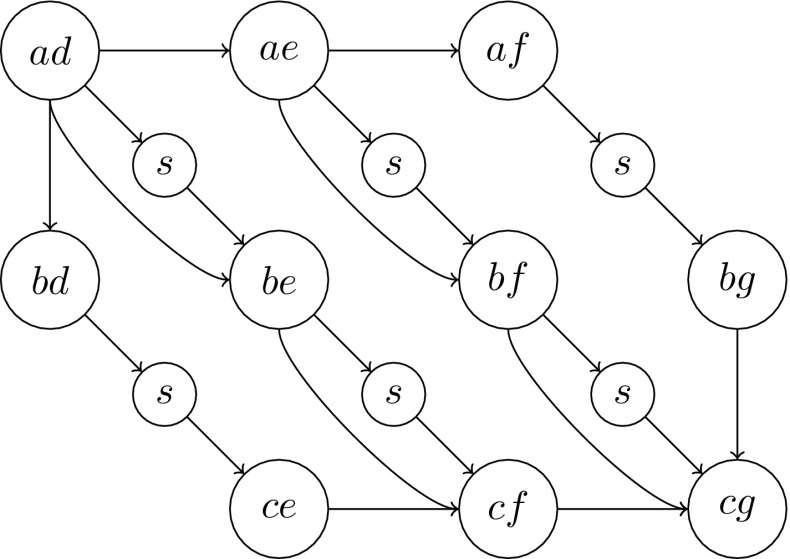
Example graph

## Appendix

### Alternative approaches for individual synchronized timing optimization

#### DPit approach

The first alternative approach relies on dynamic programming (DP). DP has been successfully employed in the vehicle routing domain within the giant tour concept (Prins 2011) and for identifying the best insertion positions of intermediate facilities (Hemmelmayr et al. 2013). In our context, the fundamental concept is the way the graph $G_{DP}$ is defined, for finding the shortest path while taking care of synchronization issues. Let $r_1$ and $r_2$ denote the routes of synchronized task *s* and let $s_1$ and $s_2$ denote the synchronized task inserted into $r_1$ and $r_2$, respectively. We propose to construct a graph of $|r_1| \times |r_2| + 1$ nodes. Each of the nodes in $G_{DP}$ corresponds to one vertex of $r_1$ and one vertex of $r_2$. If the nodes were given in a grid like layout, horizontal arcs would correspond to moving to the successor vertex in $r_2$ while staying at the same vertex in $r_1$; vertical arcs would correspond to moving to the successor vertex in $r_1$ while staying at the same vertex in $r_2$. Diagonal arcs would finally correspond to moving to the successor vertex in both routes. Diagonal nodes may also be connected via a detour through the synchronized node. Thus, each node of the graph is connected to at most four nodes: the next horizontal, the next diagonal, the next vertical and the synchronized node. However, not all four arcs are necessary at all nodes. The source node has to be connected to all four nodes. All nodes containing an origin vertex of either of the two routes have to be connected to the next node where only the vertex on the opposite route is increased, the synchronized node and the next diagonal node. There are two exceptions. The two nodes representing a combination of an origin vertex of one route and a destination vertex on the other route are not connected to any other node; they cannot be part of a feasible solution. Then, the two nodes containing one of the origin depots and the vertex just before the destination depot on the opposite route are only connected to the next diagonal node via the synchronized node. The inverse has to hold for all nodes containing the destination depot of either of the two routes. Finally, all other nodes are only connected to the next diagonal node directly and via a detour through the synchronized node and no other node.

Assume the two routes *a*–*b*–c and *d*–*e*–*f*–*g* and let again denote $s_1$ the sync task to be inserted into route *a*–*b*–*c* and $s_2$ the sync task to be inserted into route *d*–*e*–*f*–*g*. Then, the source node of the graph is node *ad* (we visit *a* on the first route and *d* on the second route). From the source node we have four options: either we go to node *bd* (we visit the next node on the first tour and we stay at the source node on route two); or we go to node *be* (we visit the next node on both routes); or we go to node *ae* (we visit the next node only on route two); finally, we may also insert the sync tasks, i.e. we go to node $s = \left\{ s_1s_2 \right\}$. The sync node *s* can only be visited between two nodes where both original vertices are increased by one position: on the way from *ad* to *be*, from *bd* to *ce* and so on. This is enforced in the label setting algorithm. The sink node of the graph is *cg* and the according graph is given in Fig. 1. The sync node is duplicated so as to illustrate all possible insertion places more clearly.

At all regular nodes in $G_{DP}$, cost and time consumptions are updated for each route individually; at the synchronized node, the beginning of service times are synchronized. We solve the shortest path problem on this graph by means of a label setting algorithm.

In our label setting algorithm, the following information is stored in each label: the node $\nu$ of the label, the time consumption of route 1 $t_{r_1}$, the time consumption of route 2 $t_{r_2}$, the costs of route 1 until the current node $c_{r_1}$, the cost of route 2 until the current node $c_{r_2}$, whether the sync node *sync* has already been visited, and a pointer to the parent label.

A new label at node *j* is generated as follows; we assume that we extend label $\eta$ on arc (*i*, *j*) to label $\eta '$ and that $r_1(i)$ gives the vertex on route 1 of node *i* and $r_2(i)$ gives the vertex on route 2 of node *i*:

18 $$\begin{aligned}&\nu (\eta ') = j,\end{aligned}$$

19 $$\begin{aligned}&t_{r_1}(\eta ') = {\left\{ \begin{array}{ll} \max (e'_{r_1(i)}, t_{r_1}(\eta ) + \tau _{r_1(i),r_1(j)}, t_{r_2}(\eta ) + \tau _{r_2(i),r_2(j)}), \\ \quad \quad \hbox {if} j \hbox {is the sync node,}\\ \max (e'_{r_1(i)}, t_{r_1}(\eta ) + \tau _{r_1(i),r_1(j)}) ,\quad \hbox {otherwise.} \end{array}\right. }\end{aligned}$$

20 $$\begin{aligned}&t_{r_2}(\eta ') = {\left\{ \begin{array}{ll} t_{r_1}(\eta '),\quad \hbox {if} \quad j \hbox {is the sync node,} \\ \max (e'_{r_2(j)}, t_{r_2}(\eta ) + \tau _{r_2(i),r_2(j)}),\quad \hbox {otherwise.} \end{array}\right. }\end{aligned}$$

21 $$\begin{aligned}&c_{r_1}(\eta ') = c_{r_1}(\eta ) + c_{r_1(i),r_1(j)} \end{aligned}$$

22 $$\begin{aligned}&c_{r_2}(\eta ') = c_{r_2}(\eta ) + c_{r_2(i),r_2(j)} \end{aligned}$$

23 $$\begin{aligned}&sync(\eta ') = {\left\{ \begin{array}{ll} 1,\quad \hbox {if} \quad j \hbox {is the sync node,} \\ sync(\eta ), \quad \hbox {otherwise.} \end{array}\right. } \end{aligned}$$

24 $$\begin{aligned}&parent(\eta ') = \text {pointer to } \eta \end{aligned}$$

The time consumption $\tau _{r_k(i),r_k(j)}$ on each arc (*i*, *j*) is computed as follows:

25 $$\begin{aligned} \tau _{r_k(i),r_k(j)}&= {\left\{ \begin{array}{ll} s_{r_k(i)} + t_{r_k(i),r_k(j)}, \text { if} r_k(i) \ne r_k(j), \\ 0,\quad \hbox {otherwise.} \end{array}\right. }&\forall k \in \left\{ 1, 2 \right\} . \end{aligned}$$

At the source node 0, $t_{r_1} = e'_{r_1(0)}, t_{r_2} = e'_{r_2(0)}, c_{r_1} = c_{r_2} = 0, sync = 0$. Then, extension of label $\eta$ on arc (*i*, *j*) is only possible if the following holds:

26 $$\begin{aligned} t_{r1}(\eta ) + \tau _{r_1(i),r_1(j)} \le l'_{r_1(j)},\end{aligned}$$

27 $$\begin{aligned} t_{r2}(\eta ) + \tau _{r_2(i),r_2(j)} \le l'_{r_2(j)}. \end{aligned}$$

In addition, in the case where $r_1(i) \in D_d$ or $r_2(i) \in D_d$, the sync node must have been visited already:

28 $$\begin{aligned} sync(\eta ) = 1. \end{aligned}$$

Finally, in the case where $\nu (\eta ) = s$ (the sync node), *i* has to be the next diagonal node:

29 $$\begin{aligned} i = { successor}[\nu (parent(\eta ))]. \end{aligned}$$

This information is stored in a *successor* vector. In order to further improve the label setting algorithm, we also use dominance rules; that is, a label is only extended if it is not dominated by any other label. Label $\eta$ dominates label $\eta '$ if the following holds:

30 $$\begin{aligned}&\nu (\eta ) = \nu (\eta '), \end{aligned}$$

31 $$\begin{aligned}&sync(\eta ) = sync(\eta '),\end{aligned}$$

32 $$\begin{aligned}&t_{r_1}(\eta ) \le t_{r_1}(\eta '), t_{r_2}(\eta ) \le t_{r_2}(\eta '), \end{aligned}$$

33 $$\begin{aligned}&c_{r_1}(\eta ) \le c_{r_1}(\eta '), c_{r_2}(\eta ) \le c_{r_2}(\eta '). \end{aligned}$$

#### CGit approach

The second alternative approach is inspired by column generation (CG). In a first step, for each of the two routes considered, all feasible insertion positions of *s* are determined and the according routes are put into two route pools $\Omega _{r_1}$ and $\Omega _{r_2}$, referring to the route pools of route $r_1$ and route $r_2$, respectively; $\Omega = \Omega _{r_1} \cup \Omega _{r_2}$. For each insertion position, we also calculate the beginning of service time $b_r$ of the synchronized task and its forward time slack $f_r$ (Savelsbergh 1992). We then solve a set partitioning problem where exactly one route has to be chosen from each of the two sets, while the beginning of service of $s_1$ and $s_2$ have to be synchronized; that is, their current beginning of service times can at most be increased by $f_r$. Let $c_r$ denote the routing costs of route *r* and $b_r$ the earliest time the synchronized task can be visited on route *r*. We use binary variables $y_r$, equal to one if route *r* is selected and zero otherwise, and continuous variables $F_r$, giving the amount of time by which the beginning of service $b_r$ has to be increased to yield synchronized service. Then, the pairwise synchronization problem can be formulated in terms of the following set partitioning type model:

34 $$\begin{aligned}&\min \sum _{r \in \Omega } c_r y_r\end{aligned}$$

35 $$\begin{aligned}&\sum _{r \in \Omega _i} y_r = 1 \quad \forall i \in \left\{ r_1,r_2 \right\} ,\end{aligned}$$

36 $$\begin{aligned}&\sum _{r \in \Omega _{r_1}}(y_r b_{r} + F_r) = \sum _{r' \in \Omega _{r_2}} (y_{r'} b_{r'} + F_{r'}),\end{aligned}$$

37 $$\begin{aligned}&0 \le F_r \le y_r f_r \quad \forall r \in \Omega ,\end{aligned}$$

38 $$\begin{aligned}&y_r \in \left\{ 0,1 \right\} \quad \forall r \in \Omega . \end{aligned}$$

The objective function (34) minimizes total routing costs. Constraints (35) make sure that exactly one route is chosen from each set $\Omega _{r1}$ and $\Omega _{r2}$. Constraint (36) guarantees pairwise route synchronization and constraints (37) limit the amount of time by which the beginning of service time at the sync node on route *r* can be increased.
